# Supplementary material for: CDKL5 Deficiency Disorder: Revealing the Molecular Mechanism of Pathogenic Variants
Source: Int J Mol Sci. 2025 Aug 29;26(17):8399. doi: 10.3390/ijms26178399 (PMC12429097; doi:10.3390/ijms26178399)
Supplement: Supplementary file 1 [file ijms-26-08399-s001.zip › 3849787_suppl_1.pdf]

Supplementary: CDKL5 Partners

A chemical genetic approach identified direct phosphorylation substrates of CDKL5: microtubule-associated protein 1S (MAP1S), rho guanino nucleotide exchange factor 2 (ARHGEF2), microtubule-associated protein RP/EB family member 2 (MAPRE2/EB2), [1]. The CDKL5 also found to phosphorylate MAPRE2/EB2 at Ser222 within the consensus motif RPX[S/T][A/G/P/S] [1]. Additionally, CDKL5 phosphorylates ARHGEF2 at Ser122 in the consensus motif RPX[S/T][A/G/P/S], a site that may influence GDP-GTP conversion in Rho GTPases [2], although its precise functions remain elucidated [1]. Another study identified Centrosomal Protein 131 (CEP131) and Disks Large Homolog 5 (DLG5) as CDKL5 phosphorylation targets. CDKL5 phosphorylated CEP131 at Ser35, a site embedded within the RPX[S/T][A/G/P/S] motif, implicating role of CDKL5 in centrosomal integrity and primary cilium assembly, consistent with established role of CEP131 in centriolar satellite organization and ciliogenesis [3].

Extending the investigation into nuclear signaling, three targets of CDKL5 were identified through a phosphoproteomic screening focused on RPX[S/T][A/G/P/S] motif-containing proteins: Elongin A (ELOA), E1A Binding Protein P400 (EP400), and Trichothiodystrophy Non-Photosensitive 1 (TTDN1) [4]. The ELOA, a transcriptional elongation factor and E3 ubiquitin ligase component, is phosphorylated at Ser311; this modification is abolished in kinase-dead CDKL5 mutants and restored by wild-type CDKL5, indicating direct regulation of RNA polymerase II elongation at DNA double-strand breaks (DSB) [2,4–6]. The EP400, a chromatin remodeling protein within the NuA4 histone acetyltransferase complex, is phosphorylated at Ser729 in a CDKL5-dependent manner, linking CDKL5 activity to transcriptional silencing and chromatin accessibility [2,4,7]. The TTDN1, implicated in transcription-coupled DNA repair and cell-cycle regulation, CDKL5 phosphorylation site was determined at Ser40; although its function remains to be validated, CDKL5-dependent modification suggests a role in nuclear stress responses, such as transcription-coupled DNA repair processes triggering CDKL5 recruitment at the DNA damage site [4,8,9].

At the synapse level, the Amphiphysin 1 (AMPH1) was identified as an endogenous substrate of CDKL5, which phosphorylates it at Ser293 within the RPX[S/T][A/G/P/S] motif [10]. This post-translational modification disrupts AMPH1 interaction with endophilin (ENDO) and dynamin (DNM), disrupting clathrin-mediated synaptic vesicle endocytosis [2,11,12]. Under physiological conditions, the proline-rich region of AMPH1 binds to the SH3 domain of endophilin to facilitate the cargo internalization. CDKL5-mediated phosphorylation significantly reduces this binding affinity, suggesting a negative regulatory role for CDKL5 in endocytic trafficking via AMPH1 phosphorylation [2,10,13].

In another recent study, Cav2.3, a voltage-gated calcium channel (encoded by CACNAE1E), has been identified through SILAC-based

phosphoproteomic screening which is phosphorylated by CDKL5 at Ser15 in mice and Ser14 in humans, within the consensus RPX[S/T][A/G/P/S] motif [13,14]. This phosphorylation modulates channel inactivation and neuronal excitability; its loss leads to Cav2.3 gain-of-function, hyperexcitability, and seizure susceptibility. Mouse models carrying the Cav2.3 S15A phosphomutant exhibit behavioral and EEG deficits that closely mirror CDD phenotypes [13].

At the excitatory synapses, CDKL5 binds to the scaffolding protein postsynaptic density (PSD-95) via its palmitoylated N-terminal region, regulating synaptic localization and dendritic spine development [15]. Netrin-G ligand-1 (NGL-1) is phosphorylated by CDKL5 at Ser631 to stabilize PSD-95 interaction and promote synaptic contact formation [16].

CDKL5 interacts with cytoskeletal regulators like IQ motif-containing GTPase-activating protein 1 (IQGAP1) and Shootin1 (SHTN1). Specifically, CDKL5 recruits IQGAP1 to actin-rich membrane protrusions via cytoplasmic linker protein 170 (CLIP170), a known actin-microtubule plus-end tracking protein (+TIP) [2,17], which plays a role in regulating dendritic morphology [18]. Although IQGAP1 contains a putative CDKL5 phosphorylation site at Ser1443, direct phosphorylation at this residue remains unconfirmed [2,19]. In parallel, CDKL5 also binds with Shootin1 (SHTN1), a brain-specific protein essential for axon formation during neuronal polarization [20]. However, while the CDKL5-SHTN1 interaction maps to the central region of SHTN1, the particular phosphorylation site has not been demonstrated yet [21].

CDKL5 phosphorylates the selective autophagy receptor p62 at T269/S272 to promote virophagy; loss of CDKL5 function impairs capsid clearance, leading to viral protein accumulation, neuronal cytotoxicity, and increased susceptibility to neurotropic viral infection [22]. In the nucleus, CDKL5 phosphorylates Mothers against decapentaplegic homolog 3 (SMAD3) to enhance its stability and support neuronal survival [23]. In contrast, CDKL5 phosphorylates the transcription factor SOX9 at Ser199 to suppress its protective role during acute renal injury, demonstrating a non-neuronal function of CDKL5 [24]. CDKL5 also phosphorylates the N-terminal domain of DNA methyltransferase 1 (DNMT1) in a DNA-dependent manner and interacts with Methyl-CpG binding protein 2 (MeCP2), though the R175S *CDKL5* variant fails to phosphorylate MeCP2, despite binding [25,26]. CDKL5-dependent phosphorylation retains Histone Deacetylase 4 (HDAC4) in the cytoplasm; loss of CDKL5 catalytic function leads to nuclear HDAC4 accumulation and impaired neuronal maturation, which can be rescued by HDAC4 inhibition [27].

References

1. Baltussen, L.L.; Negraes, P.D.; Silvestre, M.; Claxton, S.; Moeskops, M.; Christodoulou, E.; Flynn, H.R.; Snijders, A.P.; Muotri, A.R.; Ultanir, S.K. Chemical Genetic Identification of CDKL5 Substrates Reveals Its Role in Neuronal Microtubule Dynamics. *EMBO J* **2018**, *37*, e99763, doi:10.15252/embj.201899763.

2. Van Bergen, N.J.; Massey, S.; Quigley, A.; Rollo, B.; Harris, A.R.; Kapsa, R.M.I.; Christodoulou, J. CDKL5 Deficiency Disorder: Molecular Insights and Mechanisms of Pathogenicity to Fast-Track Therapeutic Development. *Biochem Soc Trans* **2022**, *50*, 1207–1224, doi:10.1042/BST20220791.
3. Graser, S.; Stierhof, Y.-D.; Lavoie, S.B.; Gassner, O.S.; Lamla, S.; Le Clech, M.; Nigg, E.A. Cep164, a Novel Centriole Appendage Protein Required for Primary Cilium Formation. *J Cell Biol* **2007**, *179*, 321–330, doi:10.1083/jcb.200707181.
4. Khanam, T.; Muñoz, I.; Weiland, F.; Carroll, T.; Morgan, M.; Borsos, B.N.; Pantazi, V.; Slean, M.; Novak, M.; Toth, R.; et al. CDKL5 Kinase Controls Transcription-Coupled Responses to DNA Damage. *EMBO J* **2021**, *40*, e108271, doi:10.15252/embj.2021108271.
5. Conaway, R.C.; Conaway, J.W. The Hunt for RNA Polymerase II Elongation Factors: A Historical Perspective. *Nat Struct Mol Biol* **2019**, *26*, 771–776, doi:10.1038/s41594-019-0283-1.
6. Weems, J.C.; Slaughter, B.D.; Unruh, J.R.; Hall, S.M.; McLaird, M.B.; Gilmore, J.M.; Washburn, M.P.; Florens, L.; Yasukawa, T.; Aso, T.; et al. Assembly of the Elongin A Ubiquitin Ligase Is Regulated by Genotoxic and Other Stresses. *J Biol Chem* **2015**, *290*, 15030–15041, doi:10.1074/jbc.M114.632794.
7. Pradhan, S.K.; Su, T.; Yen, L.; Jacquet, K.; Huang, C.; Côté, J.; Kurdistani, S.K.; Carey, M.F. EP400 Deposits H3.3 into Promoters and Enhancers during Gene Activation. *Mol Cell* **2016**, *61*, 27–38, doi:10.1016/j.molcel.2015.10.039.
8. Nakabayashi, K.; Amann, D.; Ren, Y.; Saarialho-Kere, U.; Avidan, N.; Gentles, S.; MacDonald, J.R.; Puffenberger, E.G.; Christiano, A.M.; Martinez-Mir, A.; et al. Identification of C7orf11 (TTDN1) Gene Mutations and Genetic Heterogeneity in Nonphotosensitive Trichothiodystrophy. *Am J Hum Genet* **2005**, *76*, 510–516, doi:10.1086/428141.
9. Heller, E.R.; Khan, S.G.; Kuschal, C.; Tamura, D.; DiGiovanna, J.J.; Kraemer, K.H. Mutations in the TTDN1 Gene Are Associated with a Distinct Trichothiodystrophy Phenotype. *J Invest Dermatol* **2015**, *135*, 734–741, doi:10.1038/jid.2014.440.
10. Sekiguchi, M.; Katayama, S.; Hatano, N.; Shigeri, Y.; Sueyoshi, N.; Kameshita, I. Identification of Amphiphysin 1 as an Endogenous Substrate for CDKL5, a Protein Kinase Associated with X-Linked Neurodevelopmental Disorder. *Arch Biochem Biophys* **2013**, *535*, 257–267, doi:10.1016/j.abb.2013.04.012.
11. Micheva, K.D.; Ramjaun, A.R.; Kay, B.K.; McPherson, P.S. SH3 Domain-Dependent Interactions of Endophilin with Amphiphysin. *FEBS Lett* **1997**, *414*, 308–312, doi:10.1016/s0014-5793(97)01016-8.
12. Murakami, N.; Xie, W.; Lu, R.C.; Chen-Hwang, M.-C.; Wieraszko, A.; Hwang, Y.W. Phosphorylation of Amphiphysin I by Minibrain Kinase/Dual-Specificity Tyrosine Phosphorylation-Regulated Kinase, a Kinase Implicated in Down Syndrome. *J Biol Chem* **2006**, *281*, 23712–23724, doi:10.1074/jbc.M513497200.
13. Li, C.; Liu, Y.; Luo, S.; Yang, M.; Li, L.; Sun, L. A Review of CDKL: An Underestimated Protein Kinase Family. *International Journal of Biological Macromolecules* **2024**, *277*, 133604, doi:10.1016/j.ijbiomac.2024.133604.
14. Sampedro-Castañeda, M.; Baltussen, L.L.; Lopes, A.T.; Qiu, Y.; Sirvio, L.; Mihaylov, S.R.; Claxton, S.; Richardson, J.C.; Lignani, G.; Ultanir, S.K. Epilepsy-Linked Kinase CDKL5 Phosphorylates Voltage-Gated Calcium Channel Cav2.3, Altering Inactivation Kinetics and Neuronal Excitability. *Nat Commun* **2023**, *14*, 7830, doi:10.1038/s41467-023-43475-w.
15. Zhu, Y.-C.; Li, D.; Wang, L.; Lu, B.; Zheng, J.; Zhao, S.-L.; Zeng, R.; Xiong, Z.-Q. Palmitoylation-Dependent CDKL5-PSD-95 Interaction Regulates Synaptic Targeting of CDKL5 and Dendritic Spine Development. *Proc Natl Acad Sci U S A* **2013**, *110*, 9118–9123, doi:10.1073/pnas.1300003110.
16. Ricciardi, S.; Ungaro, F.; Hambrock, M.; Rademacher, N.; Stefanelli, G.; Brambilla, D.; Sessa, A.; Magagnotti, C.; Bachi, A.; Giarda, E.; et al. CDKL5 Ensures Excitatory Synapse Stability by Reinforcing NGL-1-PSD95 Interaction in the Postsynaptic Compartment and Is Impaired in Patient iPSC-Derived Neurons. *Nat Cell Biol* **2012**, *14*, 911–923, doi:10.1038/ncb2566.

17. Barbiero, I.; Peroni, D.; Tramarin, M.; Chandola, C.; Rusconi, L.; Landsberger, N.; Kilstrup-Nielsen, C. The Neurosteroid Pregnenolone Reverts Microtubule Derangement Induced by the Loss of a Functional CDKL5-IQGAP1 Complex. *Hum Mol Genet* **2017**, *26*, 3520–3530, doi:10.1093/hmg/ddx237.
18. Swiech, L.; Blazejczyk, M.; Urbanska, M.; Pietruszka, P.; Dortland, B.R.; Malik, A.R.; Wulf, P.S.; Hoogenraad, C.C.; Jaworski, J. CLIP-170 and IQGAP1 Cooperatively Regulate Dendrite Morphology. *J Neurosci* **2011**, *31*, 4555–4568, doi:10.1523/JNEUROSCI.6582-10.2011.
19. Grohmanova, K.; Schlaepfer, D.; Hess, D.; Gutierrez, P.; Beck, M.; Kroschewski, R. Phosphorylation of IQGAP1 Modulates Its Binding to Cdc42, Revealing a New Type of Rho-GTPase Regulator. *J Biol Chem* **2004**, *279*, 48495–48504, doi:10.1074/jbc.M408113200.
20. Toriyama, M.; Shimada, T.; Kim, K.B.; Mitsuba, M.; Nomura, E.; Katsuta, K.; Sakumura, Y.; Roepstorff, P.; Inagaki, N. Shootin1: A Protein Involved in the Organization of an Asymmetric Signal for Neuronal Polarization. *J Cell Biol* **2006**, *175*, 147–157, doi:10.1083/jcb.200604160.
21. Nawaz, M.S.; Giarda, E.; Bedogni, F.; La Montanara, P.; Ricciardi, S.; Ciceri, D.; Alberio, T.; Landsberger, N.; Rusconi, L.; Kilstrup-Nielsen, C. CDKL5 and Shootin1 Interact and Concur in Regulating Neuronal Polarization. *PLoS One* **2016**, *11*, e0148634, doi:10.1371/journal.pone.0148634.
22. Thinwa, J.W.; Zou, Z.; Parks, E.; Sebti, S.; Hui, K.; Wei, Y.; Goodarzi, M.; Singh, V.; Urquhart, G.; Jewell, J.L.; et al. CDKL5 Regulates P62-Mediated Selective Autophagy and Confers Protection against Neurotropic Viruses. *J Clin Invest* **2024**, *134*, e168544, doi:10.1172/JCI168544.
23. Fuchs, C.; Medici, G.; Trazzi, S.; Gennaccaro, L.; Galvani, G.; Berteotti, C.; Ren, E.; Loi, M.; Ciani, E. CDKL5 Deficiency Predisposes Neurons to Cell Death through the Deregulation of SMAD3 Signaling. *Brain Pathol* **2019**, *29*, 658–674, doi:10.1111/bpa.12716.
24. Kim, J.Y.; Bai, Y.; Jayne, L.A.; Hector, R.D.; Persaud, A.K.; Ong, S.S.; Rojesh, S.; Raj, R.; Feng, M.J.H.H.; Chung, S.; et al. A Kinome-Wide Screen Identifies a CDKL5-SOX9 Regulatory Axis in Epithelial Cell Death and Kidney Injury. *Nat Commun* **2020**, *11*, 1924, doi:10.1038/s41467-020-15638-6.
25. Kameshita, I.; Sekiguchi, M.; Hamasaki, D.; Sugiyama, Y.; Hatano, N.; Suetake, I.; Tajima, S.; Sueyoshi, N. Cyclin-Dependent Kinase-like 5 Binds and Phosphorylates DNA Methyltransferase 1. *Biochem Biophys Res Commun* **2008**, *377*, 1162–1167, doi:10.1016/j.bbrc.2008.10.113.
26. Bertani, I.; Rusconi, L.; Bolognese, F.; Forlani, G.; Conca, B.; De Monte, L.; Badaracco, G.; Landsberger, N.; Kilstrup-Nielsen, C. Functional Consequences of Mutations in CDKL5, an X-Linked Gene Involved in Infantile Spasms and Mental Retardation. *J Biol Chem* **2006**, *281*, 32048–32056, doi:10.1074/jbc.M606325200.
27. Trazzi, S.; Fuchs, C.; Viggiano, R.; De Franceschi, M.; Valli, E.; Jedynak, P.; Hansen, F.K.; Perini, G.; Rimondini, R.; Kurz, T.; et al. HDAC4: A Key Factor Underlying Brain Developmental Alterations in CDKL5 Disorder. *Hum Mol Genet* **2016**, *25*, 3887–3907, doi:10.1093/hmg/ddw231.
